# Comprehensive HRV estimation pipeline in Python using Neurokit2: Application to sleep physiology

**DOI:** 10.1016/j.mex.2022.101782

**Published:** 2022-07-14

**Authors:** Martin G. Frasch

**Affiliations:** University of Washington, Seattle, WA, United States of America

**Keywords:** Heart rate variability, Biological oscillations, Higher order time series property estimation, Reproducible, tunable HRV computation

## Abstract

NeuroKit2 is a Python Toolbox for Neurophysiological Signal Processing. The presented method is an adaptation of NeuroKit2 to simplify and automate computation of the various mathematical estimates of heart rate variability (HRV) or similar time series. By default, the present approach accepts as input electrocardiogram's R-R intervals (RRIs) or peak times, i.e., timestamp of each consecutive R peak, but the RRIs or peak times can also stem from other biosensors such as photoplethysmography (PPGs) or represent more general kinds of biological or non-biological time series oscillations. The data may be derived from a single or several sources such as conventional univariate heart rate time series or intermittently weakly coupled fetal and maternal heart rate data. The method describes preprocessing and computation of an output of 124 HRV measures including measures with a dynamic, time-series-specific optimal time delay-based complexity estimation with a user-definable time window length. I also provide an additional layer of HRV estimation looking at the temporal fluctuations of the HRV estimates themselves, an approach not yet widely used in the field, yet showing promise (doi: 10.3389/fphys.2017.01112). To demonstrate the application of the methodology, I present an approach to studying the dynamic relationships between sleep state architecture and multi-dimensional HRV metrics in 31 subjects. NeuroKit2′s documentation is extensive. Here, I attempted to simplify things summarizing all you need to produce the most extensive HRV estimation output available to date as open source and all in one place. The presented Jupyter notebooks allow the user to run HRV analyses quickly and at scale on univariate or multivariate time-series data. I gratefully acknowledge the excellent support from the NeuroKit team.

• Univariate or multivariate time series input; ingestion, preprocessing, and computation of 124 HRV metrics.
• Estimation of intra- and inter-individual higher order temporal fluctuations of HRV metrics.
• Application to a sleep dataset recorded using Apple Watch and expert sleep labeling.

## Graphical abstract

**Figure:**
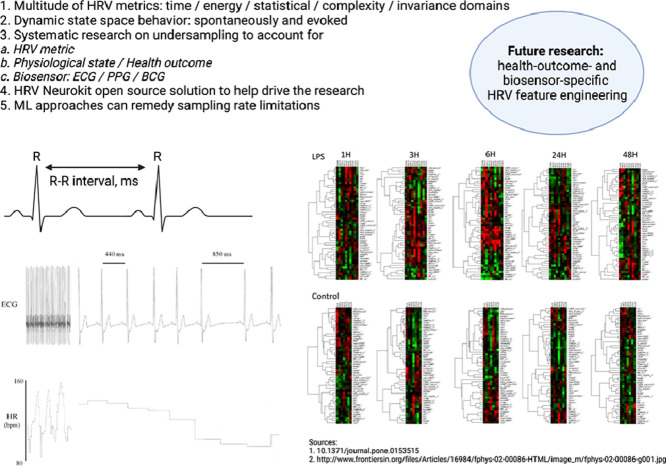

Specifications table

**Table:** 

Subject Area	Bioinformatics
More specific subject area	*Heart Rate Variability (HRV)* *Biological oscillations* *Physiology* *Time series oscillations*
Method name	*Comprehensive HRV estimation in Python*
Name and reference of original method	*The original NeuroKit2 software was described by Makowski et al. in 10.3758/s13428-020-01516-y .* *The sleep study data are available at 10.13026/hmhs-py35*
Resource availability	*The software code for the method presented here is published at* 10.5281/zenodo.5736571. The Jupyter notebook can also be accessed directly at https://github.com/martinfrasch/NeuroKit/blob/master/batch_mode_v3.1_GitHub%20_FINAL.ipynb; the datasets and updated notebook and Docker container can also be found at 10.6084/m9.figshare.20076464.v2 and https://hub.docker.com/r/mfrasch/hrv-pipeline *The GitHub repository for the underlying NeuroKit2 package is available at*https://github.com/neuropsychology/NeuroKit or at 10.5281/zenodo.3597886

## Method details

The methods section is structured as follows. First, following a brief rationale for the method I outline the HRV metrics computed. Second, I describe the implementation in Python. This section contains several elements defining the functions for executing the data loading, preprocessing and feature computation steps followed by data saving; as last step, I provide the code to tie everything together for a single step execution. At last, I present an application of the code to an open-source dataset and conclude with remarks for the broader usage.

## Introduction

Heart rate variability (HRV) as a search term on PubMed rendered ∼55,000 publications as of June 16, 2022. While first studies appeared in 1925, there has been a notable rise in scientific publishing around 1975 with some 400 papers appearing annually as of 2021. This is likely attributable to the steady increase in computational capacity and its access to it along with growing recognition of the HRV physiology and pathophysiology. For example, HRV has been recognized as a biomarker of health and stress in adult and developing organisms reflecting heart-brain interactions and resulting, among other observations, in the phenomenon of heart beat-evoked potentials, a direct reflection of bidirectional brain-heart communication [1], [2], [3], [4], [5].

The number of HRV estimates, sometimes also referred to as metrics or biomarkers, has grown as well, now exceeding 100, albeit it is understood that some of these estimates are collinear [6], [7], [8]. Still, they tend to offer unique advantages depending on the computational bottlenecks, length and noisiness of data and the desire for interpretability.

With the advent of Digital Health and increased utilization of wearable or ambient sensors to capture heart rate and other biological oscillations, the awareness of caveats in HRV analysis in contrast to the traditional electrocardiogram (ECG)-based approach also needs to rise [9]. I discuss some of the sensor-driven limitations of HRV analyses due to sampling rate in the accompanying article [10].

Several toolboxes have been built to collate the existing methodologies in a more accessible format and foster the discovery of new biomarkers of health outcomes based on HRV and other physiological time series [11], [12], [13]. Despite great advances in unification of the many features into a single software system, a major limitation has remained the reliance on commercially available environments to run it.

In parallel, the ecosystem of Python-based open-source packages for time series processing has also been maturing. One such package stands out in terms of methodological scope, functional depth, rich API and constant updates through a large international community of researchers: NeuroKit2. It is a Python Toolbox for Neurophysiological Signal Processing [14], [15], [16].

The presented method is an adaptation of NeuroKit2 to simplify and automate computation of the various mathematical estimates of HRV or similar time series [17]. By default, the present approach accepts as input electrocardiogram's R-R intervals (RRIs) or peak times, i.e., timestamp of each consecutive R peak, but the RRIs or peak times can also stem from other biosensors such as photoplethysmography (PPGs) or represent more general kinds of biological or non-biological time series oscillations. The data may be derived from a single or several sources such as conventional univariate heart rate time series or intermittently weakly coupled fetal and maternal heart rate data.

The method describes preprocessing and computation of an output of 124 HRV measures including measures with a dynamic, time-series-specific optimal time delay-based complexity estimation with a user-definable time window length (Table 1).

**Table 1:** Heart rate variability (HRV) metrics computed in the present adaptation of the NeuroKit2 Python toolbox [14,15].

Time domain [8]	RMSSD	The square root of the mean of the sum of successive differences between adjacent RR intervals. It is equivalent (although on another scale) to SD1, and therefore it is redundant to report correlations with both [20].
	MeanNN	The mean of the RR intervals.
	SDNN	The standard deviation of the RR intervals.
	SDSD	The standard deviation of the successive differences between RR intervals.
	CVNN	The standard deviation of the RR intervals (SDNN) divided by the mean of the RR intervals (MeanNN).
	CVSD	The root mean square of the sum of successive differences (RMSSD) divided by the mean of the RR intervals (MeanNN).
	MedianNN	The median of the absolute values of the successive differences between RR intervals.
	MadNN	The median absolute deviation of the RR intervals.
	MCVNN	The median absolute deviation of the RR intervals (MadNN) divided by the median of the absolute differences of their successive differences (MedianNN).
	IQRNN	The interquartile range (IQR) of the RR intervals.
	pNN50	The proportion of RR intervals greater than 50ms, out of the total number of RR intervals.
	pNN20	The proportion of RR intervals greater than 20ms, out of the total number of RR intervals.
	TINN	A geometrical parameter of the HRV, or more specifically, the baseline width of the RR intervals distribution obtained by triangular interpolation, where the error of least squares determines the triangle. It is an approximation of the RR interval distribution.
	HTI	The HRV triangular index, measuring the total number of RR intervals divided by the height of the RR intervals histogram.
	SDANN1	The standard deviation of average RR intervals extracted from n-minute segments of time series data (1, 2 and 5 by default).
	SDANN2	
	SDNNI2	The mean of the standard deviations of RR intervals extracted from n-minute segments of time series data (1, 2 and 5 by default).
	SDANN5	
	SDNNI5	
	MCVNN	MadNN/MedianNN (normalized).
	Prc20NN	
	Prc80NN	
	MinNN	
	MaxNN	
Frequency domain [8]	ULF	Ultra-low frequency band spectral power
	VLF	Very-low frequency band spectral power
	LF	Low frequency band spectral power
	HF	High frequency band spectral power
	VHF	Very high frequency band spectral power
	LFHF	LF/HF ratio
	LFn	LF normalized
	HFn	HF normalized
	LnHF	Natural logarithm transformed HF
Recurrence quantification [21]	RecurrenceRate	Recurrence rate (RR): Proportion of points that are labelled as recurrences. Depends on the radius r.
	Determinism	Determinism (DET): Proportion of recurrence points which form diagonal lines. Indicates autocorrelation.
	DeteRec	Ratio Determinism / Recurrence rate
	L	Average diagonal line length (L): Average duration that a system is staying in the same state.
	Divergence	Divergence (DIV).
	LEn	Entropy diagonal lines.
	Laminarity	Laminarity (LAM): Proportion of recurrence points which form vertical lines. Indicates the number of laminar phases (intermittency)
	TrappingTime	Trapping Time (TT) - Ratio Determinism / Recurrence rate (DET_RR)
	VMax	Longest vertical line length
	VEn	Entropy vertical lines
	W	Average white vertical line length.
	WMax	Longest white vertical line length.
	WEn	Entropy white vertical lines.
Characteristics of the Poincaré Plot Geometry	SD1	This is a measure of the spread of RR intervals on the Poincaré plot perpendicular to the line of identity. It is an index of short-term RR interval fluctuations, i.e., beat-to-beat variability. It is equivalent (although on another scale) to RMSSD, and therefore it is redundant to report correlations with both [20].
	SD2	SD2 is a measure of the spread of RR intervals on the Poincaré plot along the line of identity. It is an index of long-term RR interval fluctuations.
	SD1SD2	the ratio between short and long term fluctuations of the RR intervals (SD1 divided by SD2).
	S	Area of ellipse described by SD1 and SD2 (pi * SD1 * SD2). It is proportional to SD1SD2.
	CSI	The Cardiac Sympathetic Index, calculated by dividing the longitudinal variability of the Poincaré plot (4*SD2) by its transverse variability (4*SD1) [22].
	CVI	The Cardiac Vagal Index, equal to the logarithm of the product of longitudinal (4*SD2) and transverse variability (4*SD1) [22].
	CSI_Modified	The modified CSI obtained by dividing the square of the longitudinal variability by its transverse variability [23].
Indices of Heart Rate Fragmentation [24]	PIP	Percentage of inflection points of the RR intervals series.
	IALS	Inverse of the average length of the acceleration/deceleration segments.
	PSS	Percentage of short segments.
	PAS	Percentage of NN intervals in alternation segments.
Indices of Heart Rate Asymmetry (HRA), i.e., asymmetry of the Poincaré plot [25]	GI	Guzik's Index, defined as the distance of points above line of identity (LI) to LI divided by the distance of all points in Poincaré plot to LI except those that are located on LI.
	SI	Slope Index, defined as the phase angle of points above LI divided by the phase angle of all points in Poincaré plot except those that are located on LI.
	AI	Area Index, defined as the cumulative area of the sectors corresponding to the points that are located above LI divided by the cumulative area of sectors corresponding to all points in the Poincaré plot except those that are located on LI.
	PI	Porta's Index, defined as the number of points below LI divided by the total number of points in Poincaré plot except those that are located on LI.
	C1d	The contributions of heart rate decelerations and accelerations to short-term HRV, respectively [26].
	C1a	
	SD1d	Short-term variance of contributions of decelerations (prolongations of RR intervals) and accelerations (shortenings of RR intervals), respectively [26].
	SD1a	
	C2d	The contributions of heart rate decelerations and accelerations to long-term HRV, respectively [26].
	C2a	
	SD2d	SD2d and SD2a: long-term variance of contributions of decelerations (prolongations of RR intervals) and accelerations (shortenings of RR intervals), respectively [26].
	SD2a	
	Cd	The total contributions of heart rate decelerations and accelerations to HRV.
	Ca	
	SDNNd	Total variance of contributions of decelerations (prolongations of RR intervals) and accelerations (shortenings of RR intervals), respectively [26].
	SDNNa	
Indices of Complexity [8]	ApEn	The approximate entropy measure of HRV.
	SampEn	The sample entropy measure of HRV.
	MSE	The multiscale entropy measure of HR.
	CMSE	The composite multiscale entropy measure of HRV.
	RCMSE	The refined composite multiscale entropy measure of HRV.
	DFA	The detrended fluctuation analysis of the HR signal.
	CorrDim	The correlation dimension of the HR signal.
	optimal time delay	This metric, in seconds, provides time delay for optimal reconstruction of the underlying dynamic process [27].
	FuzzEn	Fuzzy Entropy [28].
	FuzzEnMSE	FuzzEn version of the multiscale entropy (MSE).
	FuzzEnRCMSE	FuzzEn version of the refined composite multiscale entropy (RCMSE).
	cApEn	Corrected version of ApEn [29].
	CREn	Cumulative residual entropy is an alternative to the Shannon differential entropy with several advantageous properties, such as non-negativity.
	DiffEn	Differential entropy, also referred to as continuous entropy) started as a (failed) attempt by Shannon to extend Shannon entropy. However, differential entropy presents some issues too, such as that it can be negative even for simple distributions (such as the uniform distribution).
	FI	Fisher information. The Fisher information was introduced by R. A. Fisher in 1925, as a measure of “intrinsic accuracy” in statistical estimation theory. As the Shannon entropy, it can be employed as a quality of an efficient measurement procedure, used to estimate a system's disorder [30].
	Hjorth	Hjorth Parameters are indicators of statistical properties used in signal processing in the time domain introduced by Hjorth (1970) [31]. The parameters are activity, mobility, and complexity. NeuroKit returns complexity directly in the output tuple, but the other parameters can be found in the dictionary.
	Hurst	The hurst exponent is a measure of the "long-term memory" of a time series. It can be used to determine whether the time series is more, less, or equally likely to increase if it has increased in previous steps [32].
	KFD	The Katz's Fractal Dimension is based on euclidean distances between successive points in the signal which are summed and averaged, and the maximum distance between the starting and any other point in the sample [33]. Here, fractal dimensions range from 1.0 for straight lines, through approximately 1.15 for random-walk waveforms, to approaching 1.5 for the most convoluted waveforms.
	LZC	The Lempel-Ziv complexity quantifies the regularity of the signal by scanning symbolic sequences for new patterns, increasing the complexity count every time a new sequence is detected. Regular signals have a lower number of distinct patterns and thus have low LZC whereas irregular signals are characterized by a high LZC. While often being interpreted as a complexity measure, LZC was originally proposed to reflect randomness [34].
	MSPEn	Multiscale permutation entropy. Permutation Entropy (PE) is a robust measure of the complexity of a dynamic system by capturing the order relations between values of a time series and extracting a probability distribution of the ordinal patterns. Using ordinal descriptors is helpful as it adds immunity to large artifacts occurring with low frequencies. PE is applicable for regular, chaotic, noisy, or real-world time series and has been employed in the context of EEG, ECG, and stock market time series.
	NLD	Fractal dimension (FD) of signal epochs via Normalized Length Density. NLD measures signal complexity on very short epochs durations (< 30 samples), for when continuous signal FD changes (or ‘running’ FD) are of interest. For methods such as Higuchi's FD, the standard deviation of the window FD increases sharply when the epoch becomes shorter. This NLD method results in lower standard deviation especially for shorter epochs, though at the expense of lower accuracy in average window FD.
	PEn	Permutation entropy.
	PFD	Petrosian fractal dimension: a fast method to estimate the fractal dimension of a finite sequence, which converts the data to binary sequence before estimating the fractal dimension from time series. Several variations of the algorithm exist (e.g., ‘A’, ‘B’, ‘C’ or ‘D’), primarily differing in the way the binary sequence is created.
	PLZC	Permutation Lempel-Ziv Complexity (PLZC) combines permutation and LZC. A finite sequence of symbols is first generated (numbers of types of symbols = dimension!) and LZC is computed over the symbol series.
	PSDslope	Fractal dimension via Power Spectral Density (PSD) slope [35]. Fractal exponent can be computed from Power Spectral Density slope (PSDslope) analysis in signals characterized by a frequency power-law dependence.
		It first transforms the time series into the frequency domain and breaks down the signal into sine and cosine waves of a particular amplitude that together “add-up” to represent the original signal. If there is a systematic relationship between the frequencies in the signal and the power of those frequencies, this will reveal itself in log-log coordinates as a linear relationship. The slope of the best fitting line is taken as an estimate of the fractal scaling exponent and can be converted to an estimate of the fractal dimension. A slope of 0 is consistent with white noise, and a slope of less than 0 but greater than –1, is consistent with pink noise, i.e., 1/f noise. Spectral slopes as steep as −2 indicate fractional Brownian motion, the epitome of random walk processes.
	RR	Relative Roughness is a ratio of local variance (autocovariance at lag-1) to global variance (autocovariance at lag-0) that can be used to classify different 'noises' [36,37].
	SDA	Standardized Dispersion Analysis [38]. SDA is part of a family of dispersion techniques used to compute fractal dimension. The standardized time series is divided in bins of different sizes and their standard deviation (SD) is calculated. The relationship between the SD and the bin size can be an indication of the presence of power-laws. For instance, if the SD systematically increases or decreases with larger bin sizes, this means the fluctuations depend on the size of the bins. The dispersion measurements are in units of the standard error of the mean. An FD of 1.5 indicates random data series, while values approaching 1.20 indicate 1/f scaling.
	SFD	Sevcik fractal dimension [39]. Method to calculate the fractal dimension of waveforms. Quickly measures the complexity and randomness of a signal.
	SVDEn	Singular Value Decomposition (SVD) Entropy. SVD entropy (SVDEn) can be intuitively seen as an indicator of how many eigenvectors are needed for an adequate explanation of the dataset. In other words, it measures feature-richness: the higher the SVD entropy, the more orthogonal vectors are required to adequately explain the dataset.
	SpEn	Spectral entropy treats the signal's normalized power distribution in the frequency domain as a probability distribution and calculates the Shannon entropy of it. A signal with a single frequency component (i.e., pure sinusoid) produces the smallest entropy. On the other hand, a signal with all frequency components of equal power value (white noise) produces the greatest entropy.
	WPEn	Weighted PE. The main shortcoming of traditional PE is that no information besides the order structure is retained when extracting the ordinal patterns, which leads to several possible issues [40]. The Weighted PE was developed to address these limitations by incorporating significant information from the time series when retrieving the ordinal patterns.
	ShanEn	Entropy is a measure of unpredictability of the state, or equivalently, of its average information content. Shannon entropy is one of the first and most basic measure of entropy and a foundational concept of information theory. Shannon's entropy quantifies the amount of information in a variable.
	HFD	The Higuchi's Fractal Dimension of the HR signal
Detrended Fluctuation Analysis (DFA) and Multifractal DFA [41]	DFA_alpha1	The monofractal detrended fluctuation analysis of the HR signal corresponding to short-term correlations
	MFDFA_alpha1_Width	The multifractal detrended fluctuation analysis of the HR signal corresponding to short-term correlations; the range of singularity exponents, corresponding to the width of the singularity spectrum.
	MFDFA_alpha1_Peak	
	MFDFA_alpha1_Mean	Multifractal DFA; the mean of singularity exponents.
	MFDFA_alpha1_Max	
	MFDFA_alpha1_Delta	
	MFDFA_alpha1_Asymmetry	
	MFDFA_alpha1_Fluctuation	
	MFDFA_alpha1_Increment	
	DFA_alpha2	The monofractal detrended fluctuation analysis of the HR signal corresponding to long-term correlations
	MFDFA_alpha2_Width	The multifractal detrended fluctuation analysis of the HR signal corresponding to long-term correlations the range of singularity exponents, corresponding to the width of the singularity spectrum.
	MFDFA_alpha2_Peak	
	MFDFA_alpha2_Mean	Multifractal DFA; the mean of singularity exponents
	MFDFA_alpha2_Max	
	MFDFA_alpha2_Delta	
	MFDFA_alpha2_Asymmetry	
	MFDFA_alpha2_Fluctuation	
	MFDFA_alpha2_Increment	
Quality control	segment duration, s	Logs the length of RRI period used for HRV each computation.

I also provide an additional layer of HRV estimation looking at the temporal fluctuations of the HRV estimates themselves, an approach not yet widely used in the field, yet showing promise.

Finally, I present an application of the proposed HRV estimation pipeline to an open-source dataset from PhysioNet acquired in 31 subjects during sleep using Apple Watch and enriched with expert annotation of sleep states [13,18,19].

How does this methodology add to the existing set of techniques and tools? NeuroKit2′s documentation is extensive. Here, I attempted to simplify things summarizing all the researcher needs to produce the most extensive HRV estimation output available to date as open source and all in one place. The presented Jupyter notebooks allow the user to run HRV analyses quickly and at scale on univariate or multivariate time-series data. I gratefully acknowledge the excellent support from the NeuroKit team.

The key features of the presented methodology are:

(1) Univariate or multivariate time series input; ingestion, preprocessing and computation of 62 HRV metrics.
(2) Standardization of RRI window lengths and RRI duration-specific computation of complexity estimates.
(3) Estimation of intra- and inter-individual higher order temporal fluctuations of HRV metrics.
(4) Application to a sleep dataset recorded using Apple Watch and expert sleep labeling.

The step-by-step approach is as follows.

1. Create a dedicated virtual environment

You may use conda or another environment manager such as pip or Docker. The choice boils down to your preferences and constraints: for example, certain Python packages can only be installed in pip and not in conda. For the proposed approach, I am not aware of any constraints that prevent the user from using conda. Ultimately, using a virtual environment will help you down the road to ensure your Python analytical pipeline keeps on working and does not get broken by unintended package updates and disrupted interdependencies. As an alternative to this conda step, I provide a Docker container here [42].

# call conda datanalysis environment

!conda init bash

!conda activate datanalysis #or use your own preferred venv

2. Load the required and recommended packages.

import neurokit2 as nk

import pandas as pd

import matplotlib.pyplot as plt

import numpy as np

import os

import scipy.io

from pathlib import Path

from scipy.stats import variation

from hmmlearn import hmm

# load Matlab data files

from scipy.io import loadmat

3. Import raw peaks.

The source may be Matlab files or whatever input data format you may need. In the present example, we load a duo of files: corresponding maternal and fetal peak data. Your use case may differ. For example, you may load just one set of peak data, three or more sets of peak data derived from different ECG channels, an ECG-derived peak times channel and a PPG-derived peak times channel, etc. Simply amend the code accordingly by editing and adding the additional lines for each step as required.

In this work, because the focus is on peak times or heart rate (or, conversely, the RRI) time series, an important step is skipped deliberately: the derivation of the peak data from the raw signal. This can be ECG, PPG or otherwise recorded blood pressure fluctuations (pulse). The HRV Task Force recommends checking for the presence of ectopic heartbeats, e.g., premature ventricular contractions (PVCs) [6,43]. NeuroKit provides an API for detecting R peaks and for artifact correction. I refer the interested reader to their documentation.

# Import raw peaks from the mat files; adjust to fit your input data format

f_filepath_peaks=Path.cwd()/"raw_peaks/f" #fetal raw peaks mat files;

m_filepath_peaks=Path.cwd()/"raw_peaks/m" #maternal raw peaks mat files;

4. Get ready for batch file processing. a. Create a list of relevant files in directory

f_peaks_files = [f for f in sorted(f_filepath_peaks.iterdir()) #create a list of relevant files in directory

if f.suffix == '.mat']

m_peaks_files = [f for f in sorted(m_filepath_peaks.iterdir()) #create a list of relevant files in directory

if f.suffix == '.mat'] b. Read one file at a time using the above list, trim, clean, convert to RRI c. The present syntax is for a specific ECG format; adopt to your use case d. Iterate over i files in the f_ or m_ peaks_files lists and extract the correct peaks channel as numpy array

def read_mat_file(f_peaks_file, m_peaks_file):

# Import 5th row of the mat file's peak data which has 1000 Hz sampling rate; you may need to adopt this step as per your data structure

f_file_PEAK_raw=loadmat(f_peaks_file)

m_file_PEAK_raw=loadmat(m_peaks_file)

f_peaks=f_file_PEAK_raw['fetal_Rpeaks'][4] #this is my 5th row ECG-SAVER-extracted peaks channel

m_peaks=m_file_PEAK_raw['mother_Rpeaks'][4] #this is my 5th row

# Trim trailing zeros

f_peaks_trimmed=np.trim_zeros(f_peaks,trim='b')

m_peaks_trimmed=np.trim_zeros(m_peaks,trim='b')

# Artifact removal [see next section for details]

f_clean_peaks=nk.signal_fixpeaks(f_peaks_trimmed, sampling_rate=1000, iterative=False, show=False,interval_min=0.33,interval_max=0.75, method="kubios") #allow 80–180 bpm

m_clean_peaks=nk.signal_fixpeaks(m_peaks_trimmed, sampling_rate=1000, iterative=False, show=False,interval_min=0.4,interval_max=1.5, method="kubios") #allow 40–150 bpm

# Document artifacts from each run as clean_peaks_rri[0]: build a dataframe for each file over all segments

# Convert to RRI

f_rri = peaks_to_rri(f_clean_peaks[1], sampling_rate=1000, interpolate=False)

m_rri = peaks_to_rri(m_clean_peaks[1], sampling_rate=1000, interpolate=False)

return f_clean_peaks[1], m_clean_peaks[1], f_rri, m_rri, f_clean_peaks[0], m_clean_peaks[0] e. Proceed with the steps below: HRV compute, save. Cf. final section (10).

5. **Convert** peaks to RRIs

Using NeuroKit2’s functions to take the cleaned peaks as input: peaks_to_rri

# Some NK functions [clean peaks function, complexity HRV metrics] take RRIs

# So use these UDFs borrowed from the NK package: convert peaks to RRI on the cleaned peaks output

def peaks_to_rri(peaks=None, sampling_rate=1000, interpolate=False, **kwargs):

rri = np.diff(peaks) / sampling_rate * 1000

if interpolate is False:

return rri

else:

# Minimum sampling rate for interpolation

if sampling_rate < 10:

sampling_rate = 10

# Compute length of interpolated heart period signal at requested sampling rate.

desired_length = int(np.rint(peaks[-1]))

rri = signal_interpolate(

peaks[1:], # Skip first peak since it has no corresponding element in heart_period

rri,

x_new=np.arange(desired_length),

**kwargs

)

return rri, sampling_rate

6. Artifact correction. a. This is a key step that will influence everything downstream. It is often not reported clearly in the studies. b. Adjust the sampling rate and threshold settings as appropriate for your data. c. Note that we save the logs of artifact correction for audit purposes. Sometimes, you need to know why a certain dataset behaved in the way it did and this documentation can come in handy.

# Artifact correction

# Integrated into the above UDF red_mat_file, but you may find this useful to adopt elsewhere in your code

https://neurokit2.readthedocs.io/en/latest/functions.html#neurokit2.signal.signal_fixpeaks

# Artifact removal on peaks using Kubios: write into UDF taking trimmed_peaks input

# caution: nk.signal_fixpeaks takes peaks, not RRI!

# nk.signal_fixpeaks saves the corrected peak locations to the [1] index of the output data sturcture

# accessible like so: clean_peaks[1]

# Review the settings for fetal versus maternal RRI inputs! Adjust to match your RRI physiology

# interval_min – minimum interval btw peaks | interval_max – maximum interval btw peaks.

f_clean_peaks=nk.signal_fixpeaks(f_peaks_trimmed, sampling_rate=1000, iterative=False, show=False,interval_min=0.1,interval_max=0.25, method="kubios")

m_clean_peaks=nk.signal_fixpeaks(m_peaks_trimmed, sampling_rate=1000, iterative=False, show=False,interval_min=0.1,interval_max=0.25, method="kubios")

# Convert trimmed and cleaned peaks to RRI (using _trimmmed_ raw peaks as input!)

rri_clean = peaks_to_rri(clean_peaks_peaks[1], sampling_rate=1000, interpolate=False)

7. Compute all HRV metrics segment-wise a. Rather than computing on the entire time series at once and trading the reproducibility as a result (HRV metrics have variable dependence on the duration of time series on which they are computed, among other things), we i. set the segment duration explicitly a priori and ii. Take advantage of the segment-wise estimate of HRV (or variability in general, as your case may be) to investigate the higher-order structure of the HRV metrics themselves. b. For complexity estimates, note that we use segment duration-specific estimation of optimal time delay rather than using default settings. This allows us to compute FuzzEn, FuzzEnMSE, FuzzEnRCMSE, cApEn specifically for the optimal time delay. Why select these complexity estimates? It is heuristic. I have found Fuzzy Entropy estimates to be understudied and robust, especially with RRI time series. This is hence worthy of additional attention in future studies deploying complexity estimates. Other time-delay-dependent complexity estimates can be plugged in here, all made available via NeuroKit2 API.

# UDF compute_HRV

# This UDF computes all [regular and extra non-linear] HRV metrics segment-wise for a file

def compute_HRV(peaks,rri,SubjectID):

# Regular HRV matrix (from peaks)

duration_peaks=peaks[len(peaks)-1] #gives me the duration in samples

divider=duration_peaks/1000/60/5 #sampling_rate, 5 min window segments

segment=np.array_split(peaks,divider) #divide in segments of 5 min; the last segment may be shorter; discard during statistical analysis on HRV metrics

segment_df=pd.DataFrame()

for i in range(len(segment)):

segment=nk.hrv(segment[i],sampling_rate=1000, show=False)

segment_df = pd.concat([segment_df,segment],ignore_index=True)

# Additional nonlinear HRV metrics from RRIs

segment=np.array_split(rri,divider) #divide _RRI_ in segments of 5 min; the last segment may be shorter; discard during statistical analysis on HRV metrics

#create my dataframe structure to which to append the list as a row in the following

extra_columns=['optimal time delay','FuzzEn','FuzzEnMSE','FuzzEnRCMSE','cApEn','segment duration, s','SubjectID']

extra_complexity_df=pd.DataFrame(columns=extra_columns)

df_length=len(extra_complexity_df)

extra_complexity_df_total=pd.DataFrame(columns=extra_columns)

for i in range(len(segment)):

optimal_complexity_parameters = nk.complexity_delay(segment[i], delay_max=100, method='fraser1986′, show=False)

extra_complexity_segment_fuzen=nk.entropy_fuzzy(segment[i], delay=optimal_complexity_parameters)

extra_complexity_segment_fuzen_mse=nk.complexity_fuzzymse(segment[i],fuzzy=True)

extra_complexity_segment_fuzen_rcmse=nk.complexity_fuzzyrcmse(segment[i], fuzzy=True, composite=True, refined=True)

extra_complexity_segment_capen=nk.entropy_approximate(segment[i], delay=optimal_complexity_parameters, corrected=True)

segment_duration=np.sum(segment[i])/1000 #segment duration in seconds

#join all individual output floats including values of segment[i] - i.e., for each segment - and its duration in seconds as numpy.sum(segment[1])/1000

extra_complexity = [optimal_complexity_parameters, extra_complexity_segment_fuzen,extra_complexity_segment_fuzen_mse,extra_complexity_segment_fuzen_rcmse,extra_complexity_segment_capen,segment_duration,SubjectID]

extra_complexity_df.loc[df_length]=extra_complexity

extra_complexity_df_total = pd.concat([extra_complexity_df_total,extra_complexity_df],ignore_index=True)

# simply concatenate both df's horizontally; this scales allowing addition of other df's from bivariate computations

final_df=pd.concat([segment_df, extra_complexity_df_total],axis=1)

return final_df #this is per subject with SubjectID output along on the right side

8. Compute higher order HRV metrics.

Here I made explicit and expanded upon what we attempted first in [44] a. First, basic variability statistics are defined. b. Next, a hidden markov model (HMM) is implemented. c. Finally, everything is put together and saved. d. Note that I left here a number of commented lines of code for future development. I welcome improvements on those! For example, HMM code requires a certain duration of HRV metrics time series to compute. Since in the original dataset where this method was developed, the number of datapoints was limited to 6–8, I commented it out and made it available here for future reference and use on larger datasets.

def compute_basic_stats(ts_data, SubjectID):

# compute mean and variation

# assuming "ts_data" is where my HRV metric values list is per subject

mean=np.mean(ts_data.values.tolist())

coeff_variation=variation(ts_data.values.tolist())

# this function works similar to variation() but works purely with numpy

# cv = lambda x: np.std(x) / np.mean(x)

# First quartile (Q1)

Q1 = np.percentile(ts_data, 25, interpolation = 'midpoint')

# Third quartile (Q3)

Q3 = np.percentile(ts_data, 75, interpolation = 'midpoint')

# Interquaritle range (IQR)

IQR = Q3 - Q1

midhinge = (Q3 + Q1)/2

quartile_coefficient_dispersion = (IQR/2)/midhinge

# adding entropy estimate; this is experimental!

# ts_entropy=nk.entropy_sample(ts_data)

# yielding error "could not broadcast input array from shape (7,1) into shape (7)" | the following syntax fixes that and is more elegant in that it estimates optimal delay

# optimal_complexity_parameters = nk.complexity_delay(ts_data.to_numpy, delay_max=6, method='fraser1986′, show=False)

# ts_entropy=nk.entropy_fuzzy(ts_data.to_numpy, delay=optimal_complexity_parameters)

# still yielding len error

ts_entropy=nk.entropy_shannon(ts_data)

basic_stats=[SubjectID, mean, coeff_variation[0], quartile_coefficient_dispersion, ts_entropy]

return basic_stats

#HMM Model

def do_hmm(ts_data):

#ts_data=numpy.array(data)

gm = hmm.GaussianHMM(n_components=2)

gm.fit(ts_data.reshape(-1, 1))

hmm_states = gm.predict(ts_data.reshape(-1, 1))

#hmm_states=[states.tolist()]

print(hmm_states)

return hmm_states # next, add _states_ iteratively for all subjects to states_Uber list to spot patterns

# deal with last column which is string and needs to be skipped

def skip_last_column(lst):

# unpack the list of lists

def Extract(lst):

return [item[0] for item in lst]

# check for string in the first sublist (all I need to decide to skip it for numpy operations)

element_to_check=Extract(lst)[0]

return isinstance(element_to_check, str) #return Boolean for presence of string in the sublist

def compute_higher_HRV(final_df, SubjectID):

# assuming "final_df" is the dataframe where the HRV metric values are listed segment-wise per subject

# compute basic stats

higher_order_basic_stats=[]

for i in range(final_df.shape[1]): #last column is the SubjectID string, so skipping it below

metric=final_df.iloc[:,[i]].values

#String skip logic to skip over SubjectID column

if skip_last_column(final_df.iloc[:,[i]].values) == False:

results_temp1=compute_basic_stats(final_df.iloc[:,[i]].astype(np.float64),SubjectID)

higher_order_basic_stats.append(results_temp1)

else:

i+=1

basic_stats=pd.DataFrame(higher_order_basic_stats, columns=['SubjectID','mean', 'coeff_variation', 'quartile_coefficient_dispersion','HRV metrics entropy'])

columns=final_df.columns[0:63] #make sure I don't select the last column which has SubjectID

basic_stats.index=[columns]

basic_stats_final=basic_stats.T #transpose

# compute HMM stats: computing on just 7 data points leads to errors in some instances, so omit for now and revisit later when used on longer HRV metrics time series, say, several hours

# Estimate HMM probabilities output for a given segmented HRV metric

# Then compute basic_stats on this estimate;

# Hypothesis: stable tracings will have tight distributions of HMM values and resemble entropy estimates;

# This will apply statistically significantly for physiologically stressed (tighter distributions) versus control subjects

#higher_order_basic_stats_on_HMM=[]

#for i in range(final_df.shape[1]): #last column is the SubjectID string, so removing it

# metric=final_df.iloc[:,[i]].values

# print("metric has the type", type(metric))

# some HRV metrics have NaNs and the "do_hmm" script crashes on those;

# Adding logic to skip if NaN is present

# a=any(pd.isna(metric)) #checking if _any_ values in HRV metrics list are NaN

# b=skip_last_column(metric)

# skip_reasons={a:'True', b:'True'}

#NaN or string skip logic

# if any(skip_reasons):

# i+=1

# else:

# results_hmm_temp2=do_hmm(metric)

# print(results_hmm_temp2)

# print(type(results_hmm_temp2))

# results_stats_hmm_temp=compute_basic_stats(results_hmm_temp2.tolist(),SubjectID) #j being the file number; != SubjectID

# higher_order_basic_stats_on_HMM.append(results_stats_hmm_temp)

#basic_stats_on_HMM=pd.DataFrame(higher_order_basic_stats_on_HMM, columns=['HMM_mean', 'HMM_coeff_variation', 'HMM_quartile_coefficient_dispersion','HMM_HRV metrics entropy'])

#basic_stats_on_HMM.index=[columns]

#basic_stats_on_HMM_final=basic_stats_on_HMM.T #transpose

#higher_final_df=pd.concat([basic_stats_final, basic_stats_on_HMM_final],axis=1)

higher_final_df=basic_stats_final #leaving the syntax above for when the data allow HMM analysis

return higher_final_df #this includes SubjectID

9. Save everything.

Gather all data from the separate data frames into spreadsheets for further analyses.

# Execute the entire analysis

For each file (fetal and maternal):

- call read_mat_file

- call compute_HRV

- save results to Excel

10. Execute the entire pipeline calling the above defined functions

# Initialize data structures

f_artifacts_log=[]

m_artifacts_log=[]

Uber_fHRV=[]

Uber_mHRV=[]

Uber_higher_fHRV=[]

Uber_higher_mHRV=[]

i=0

# Compute & save into lists

while i<=len(f_peaks_files)-1: #careful - this assumes equal number of fetal and maternal raw files

# read the peaks file, trim trailing zeros, artifact correct it, convert to RRIs and return the results

f_clean_peaks, m_clean_peaks, f_rri, m_rri, f_clean_peaks_artifacts, m_clean_peaks_artifacts=read_mat_file(f_peaks_files[i],m_peaks_files[i])

fSubjectID=format(f_peaks_files[i].stem)

mSubjectID=format(m_peaks_files[i].stem)

f_artifacts_log_i=[fSubjectID,f_clean_peaks_artifacts]

m_artifacts_log_i=[mSubjectID,m_clean_peaks_artifacts]

#save artifact processing log from each file starting with its real SubjectID

f_artifacts_log.append(f_artifacts_log_i)

m_artifacts_log.append(m_artifacts_log_i)

# compute all HRV metrics

ffinal=compute_HRV(f_clean_peaks,f_rri,fSubjectID)

mfinal=compute_HRV(m_clean_peaks,m_rri,mSubjectID)

# update the UBER df

Uber_fHRV.append(ffinal)

Uber_mHRV.append(mfinal)

# compute higher_order HRV metrics

fhigher_final=compute_higher_HRV(ffinal,fSubjectID)

mhigher_final=compute_higher_HRV(mfinal,mSubjectID)

# update the UBER_higher_df

Uber_higher_fHRV.append(fhigher_final)

Uber_higher_mHRV.append(mhigher_final)

i+=1

if i>len(f_peaks_files):

break

print('Computation completed.')

# save artifacts logs

df_Uber_f_artifacts = pd.DataFrame.from_records(f_artifacts_log) #edit the name as needed

df_Uber_m_artifacts = pd.DataFrame.from_records(m_artifacts_log) #edit the name as needed

df_Uber_f_artifacts.to_excel('analysis/fUBER_artifacts_log.xlsx', index=False)

df_Uber_m_artifacts.to_excel('analysis/mUBER_artifacts_log.xlsx', index=False)

# save HRV results

Uber_fdf=pd.concat(Uber_fHRV)

Uber_fdf.to_excel("analysis/fmetrics.xlsx")

Uber_mdf=pd.concat(Uber_mHRV)

Uber_mdf.to_excel("analysis/mmetrics.xlsx")

Uber_higher_fdf=pd.concat(Uber_higher_fHRV)

Uber_higher_fdf.to_excel("analysis/higher_fmetrics.xlsx")

Uber_higher_mdf=pd.concat(Uber_higher_mHRV)

Uber_higher_mdf.to_excel("analysis/higher_mmetrics.xlsx")

11. Method validation: Demonstrating the performance of the proposed HRV pipeline in a retrospective analysis of a polysomnography dataset recorded with Apple Watch.

As validation dataset, the data by Walch et al. was used which is available from PhysioNet. The team acquired heart rate data in 31 subjects during sleep using Apple Watch and enriched the data with expert annotation of sleep states [13,18,19]. The labeled sleep state architecture was recorded from polysomnography and saved in the format '[subject-id-number]_labeled_sleep.txt'. Each line in this file has the format: *date (in seconds since polysomnography start) stage (0-5, wake = 0, N1 = 1, N2 = 2, N3 = 3, REM = 5).*

This dataset is appealing for several reasons for the intended objective of HRV pipeline validation:

(1) The heart rate data, extracted from the Apple watch, is publicly available.
  The data were recorded from 31 subjects during sleep, averages 7.3 h, and comes expertly annotated with sleep state labels.
(2) The authors provided a script on GitHub for how to enable such data extraction in the future. This should make such a demonstration particularly relevant for future studies [45].
(3) This dataset ties in well with the accompanying publication in the Journal of Biomedical Informatics [10] where I discuss the impact of sampling rate on HRV estimation. The Apple Watch data are a good example of the potential of wearables to provide physiological insights which are fundamentally limited by low sampling rates of the underlying signal used to derive HRV (PPG in this case).

Interestingly, the presented HRV pipeline yields insights into sleep state dynamics reflected in HRV which I discuss below. The underlying code, based on the presented HRV estimation pipeline, and all generated data can be found on FigShare and DockerHub [46].

To analyze this dataset, I expanded the presented HRV pipeline further and deployed it in several ways that can be used as a basis for future studies as follows.

**Fig. 1:**
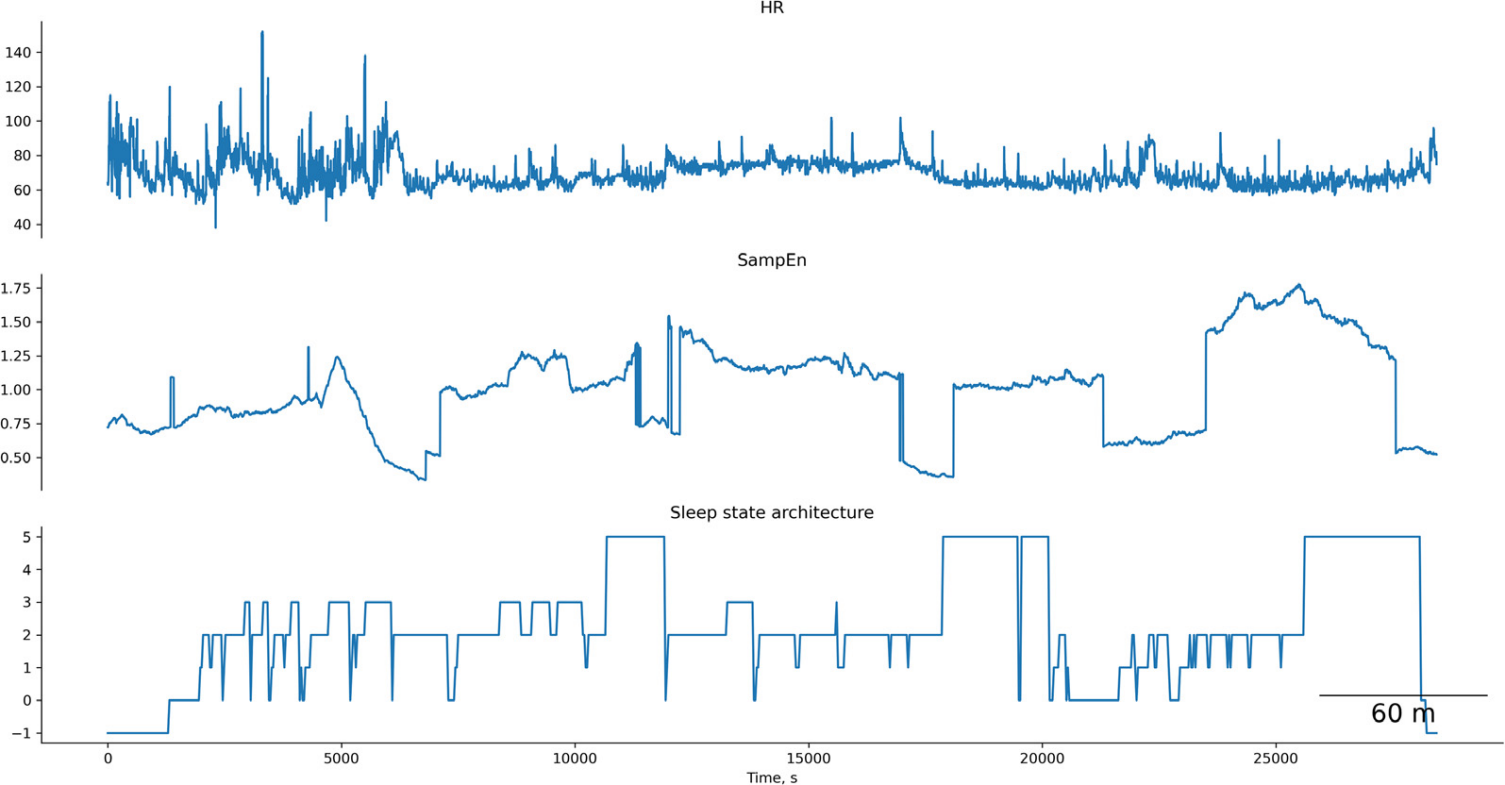
Example of the temporal relationship between Sample Entropy of HRV and sleep states computed from Apple Watch [46].
  ○ Next, as an exploratory step, Spearman correlations were computed across all subjects between HRV metrics SampEn and RMSSD on one hand and the NREM sleep state N3 (the deepest sleep state). The findings show again, this time quantitatively across the cohort, a degree of correlation between HRV complexity fluctuations and duration of deep sleep (Fig. 2). Note the correlation between N3 NREM duration and CV SampEn (*R* = 0.39, *p* = 0.03) or CV RMSSD (*R* = 0.55 and *p* = 0.001), respectively (Fig. 3).

• The number of HRV metrics was increased from 63 (as per above Jupyter notebook) to 124 HRV metrics, computed on this entire PhysioNet dataset (cf. Table 1).
• Since this is intended as an example only, for ease of computing, I used the entire dataset to compute HRV (i.e., divider = 1 rather than performing the sliding window computations); I also set optimization for complexity parameters to default settings for the same reasons. The code is available for those who wish to dive deeper and have the resources to do so.
• Sample Entropy (SampEn) is reported as an example complexity metric of HRV over time as it changes during sleep along with the traditional linear time-domain metric RMSSD; these are plotted along with the heart rate and sleep state architecture (using the supplied labels). This approach can contribute to studying these relationships systematically and develop open source algorithms to reliably detect sleep states from PPG-derived HRV data.
  ○ The code is presented to determine the total duration of each sleep state per recording using the labeled files [46].
  ○ The saved continuous and averaged SampEn and RMSSD data are provided for the entire cohort, for future analyses [46].
  ○ The code and the visualizations of each subject's time course are provided for heart rate, SampEn, and sleep state architecture [46]. These data reveal a certain covariance between the HRV complexity and sleep state dynamics (Fig. 1).

**Fig. 2:**
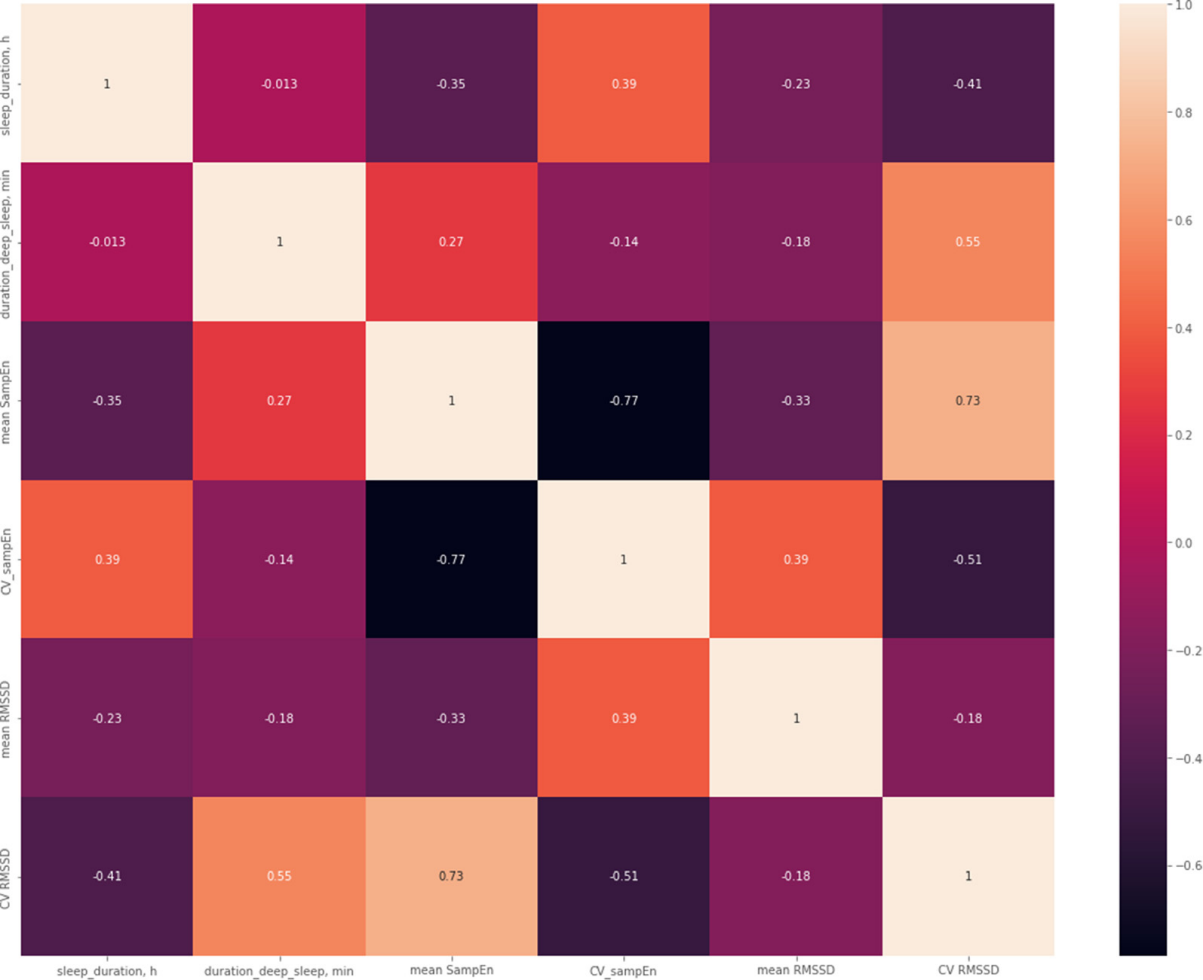
Spearman correlations between the durations of N3 stage of NREM sleep and HRV complexity metric SampEn as well as the linear time domain metric RMSSD. As a representative metric of higher-order variability, the temporal variability, gauged as coefficient of variation (CV), of these two HRV metrics is also considered [44,47].

**Fig. 3:**
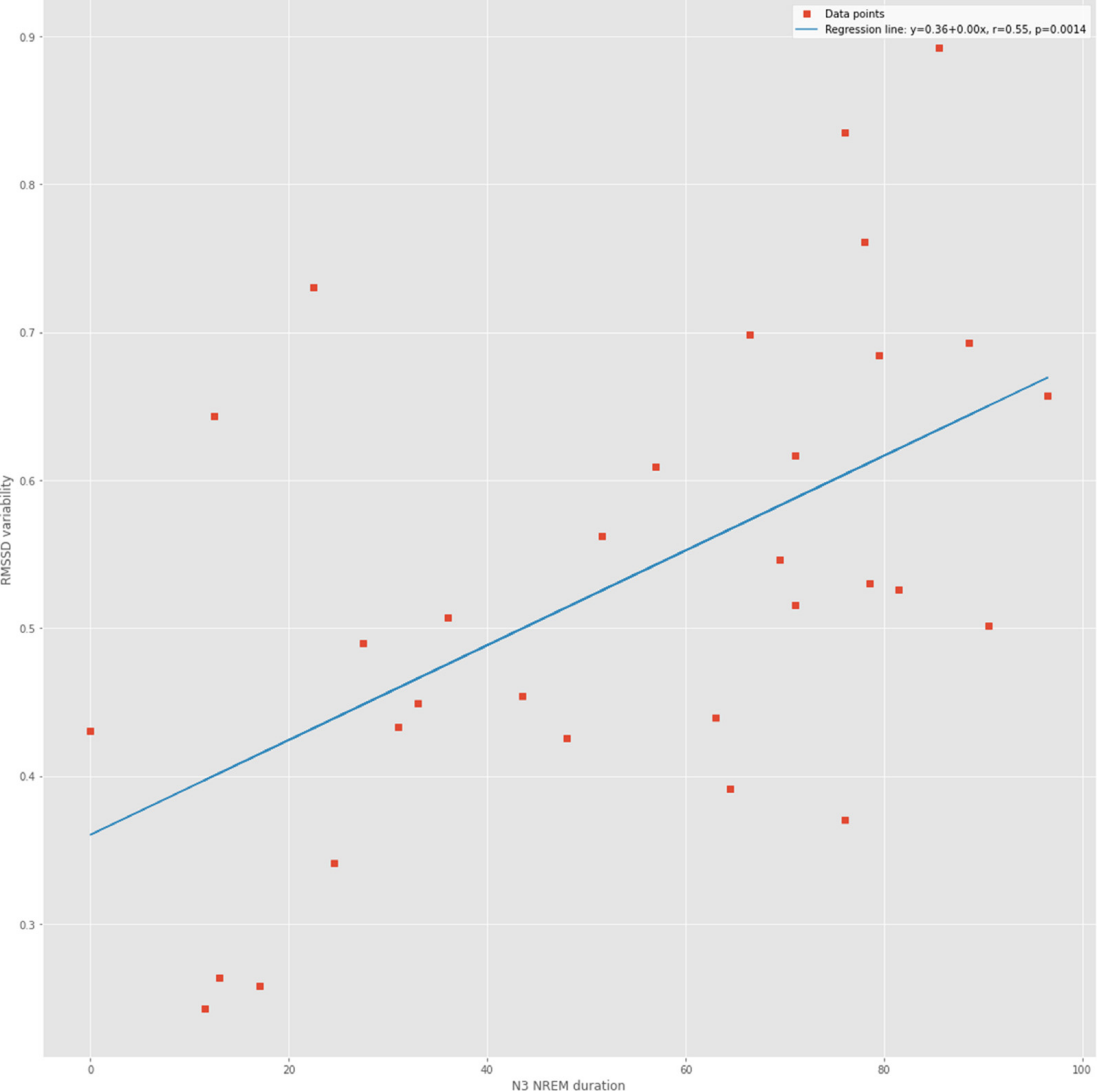
The variability of RMSSD correlates with N3 NREM duration.
  ○ Next, I expanded the scope by assessing and showing the correlations systematically for all subjects, all 124 HRV metrics, and all sleep states (all code and results provided) (Fig. 4) [46].

**Fig. 4:**
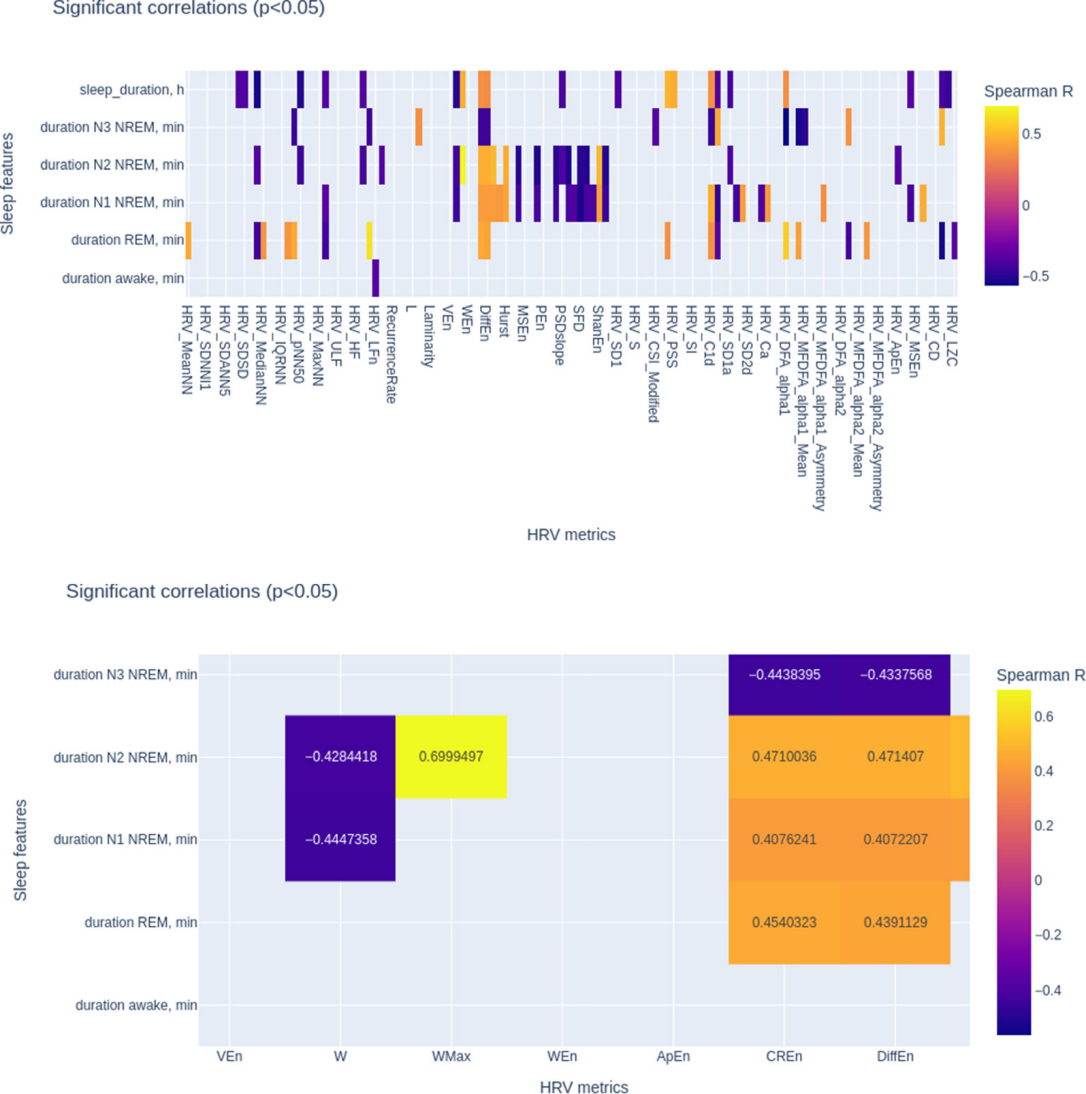
Correlations between sleep state durations and HRV metrics. TOP: all metrics and sleep states are shown for which Spearman R values were found where *p* < 0.05. BOTTOM: A selective zoom on the strong correlations. See Table 1 for HRV metrics legend. The resulting dynamic visualization of correlations between HRV metrics and sleep states with Plotly can be viewed here: https://plotly.com/~mfrasch/5/import-pandas-as-pd-import-plotlyexpres/.

I suggest the following implications for future work. First, the dataset and the presented findings can be studied further using machine learning tools to derive an optimal HRV metric-based predictor of the NREM states or REM states’ duration. Second, the richness of the temporal fluctuations can be further harnessed for classification and prediction using the code for hidden Markov mechanisms (HMM). I consider this to be out of scope for the present manuscript but provide the necessary code.

12. Data availability

• All data produced during this analysis has been deposited in FigShare at 10.6084/m9.figshare.20076464 [46]. The resulting Docker container is published on https://hub.docker.com/r/mfrasch/hrv-pipeline. The notebook is deposited on GitHub pages (https://martinfrasch.github.io): for viewing online here (https://martinfrasch.github.io/MethodsX%20R1%20HRV%20pipeline%20v4.1.html)
• and as downloadable Jupyter notebook here (https://martinfrasch.github.io/MethodsX%20R1%20HRV%20pipeline%20v4.1%20FINAL.ipynb)
• The resulting dynamic visualization of correlations between HRV metrics and sleep states with Plotly can be viewed here: https://chart-studio.plotly.com/∼mfrasch/5
  The underlying data (Spearman R values for p<0.05) can be found here (https://chart-studio.plotly.com/∼mfrasch/6)

Final remarks

The presented HRV computation pipeline in Python using the API of NeuroKit2 package is shown based on the use of the maternal-fetal trans-abdominally derived non-invasive ECG signal followed by maternal and fetal ECG extraction using SAVER algorithm [48]. Therefore, two RRI inputs are coded throughout the pipeline. However, the number of inputs can vary depending on your use case from univariate RRI time series to multivariate RRI time series. As such, this approach is easily scalable to a given scenario. As an example, the approach to a univariate heart rate analysis in relation to sleep state architecture is also presented.

Recent advances in the in silico modeling of physiological systems open avenues for discovery of novel and deeper understanding of the existing HRV metrics [54], [55], [56].

The literature indicates a high potential of HRV biomarkers to serve as predictors of important health outcomes such as cardiac or mental health as well as in critical care and disorders of consciousness [1,2,6,[49], [50], [51], [52], [53]].

## Declaration of Competing Interest

The author declares the following financial interests/personal relationships which may be considered as potential competing interests:

MGF holds patents on EEG and ECG processing. MGF is founder of and consults for Digital Health companies commercializing predictive potential of physiological time series for human health.

## Data Availability

Comprehensive heart rate variability estimation in relation to sleep state architecture: a retrospective observational cohort study on Apple Watch heart rate data (Original data) (Figshare).
